# N,N Dimethylacetamide a drug excipient that acts as bromodomain ligand for osteoporosis treatment

**DOI:** 10.1038/srep42108

**Published:** 2017-02-08

**Authors:** Chafik Ghayor, Bebeka Gjoksi, Jing Dong, Barbara Siegenthaler, Amedeo Caflisch, Franz E. Weber

**Affiliations:** 1 Oral Biotechnology & Bioengineering, Center for Dental Medicine/MKG, University of Zürich, Switzerland; 2 Zurich Center for Integrative Human Physiology (ZIHP), University of Zurich, Switzerland; 3 Department of Biochemistry, University of Zurich, Switzerland; 4 CABMM, Center for Applied Biotechnology and Molecular Medicine, University of Zurich, Zurich, Switzerland

## Abstract

N,N-Dimethylacetamide (DMA) is a water-miscible solvent, FDA approved as excipient and therefore widely used as drug-delivery vehicle. As such, DMA should be devoid of any bioactivity. Here we report that DMA is epigenetically active since it binds bromodomains and inhibits osteoclastogenesis and inflammation. Moreover, DMA enhances bone regeneration *in vivo*. Therefore, our *in vivo* and *in vitro* data reveal DMA’s potential as an anti-osteoporotic agent via the inhibition of osteoclast mediated bone resorption and enhanced bone regeneration. Our results highlight the potential therapeutic benefits of DMA and the need for reconsideration of previous reports where DMA was used as an ‘inactive’ drug-delivery vehicle.

Heritable changes in gene expression or cellular phenotype not linked to changes in the underlying DNA sequence are studied in epigenetics. Such changes include acetylation known to act as scaffolds for the assembly of macromolecular complexes that orchestrate chromatin accessibility to transcription factors and allow the recruitment and activation of the RNA polymerases. The discovery of the drugability of bromodomains (BRD)^1^ has fueled the development of small molecule inhibitors. Proteins that contain bromodomains are involved in the regulation of transcriptional programs and have been identified in oncogenic rearrangements that lead to highly oncogenic fusion proteins, which have a key role in the development of several aggressive types of cancer^2^. High affinity micro-molar range bromodomain inhibitors I-BET and JQ1 were developed and presented efficiency *in vivo* against LPS-induced endotoxic shock, bacteria-induced sepsis^3^, decrease tumor size, and enhanced survival in testis midline carcinoma and myelomas^4^.

A link between bromodomains and the immune system has been shown for Brd4 and NFĸB with its two subunits p50 and RelA^5^. The latter subunit, if acetylated, binds Brd4, which in turn enhances the transcriptional activation of NF-ĸB and the expression of a subset of NF-ĸB-responsive inflammatory genes. Brd2 and Brd4 together with Brd3 and BrdT form the BET protein family of transcriptional regulators. BET proteins possess dual, mutually-related Bromodomains in the amino-terminal region and protein–protein interaction domains for association with transcription machinery in the carboxyl-terminal region, called extra terminal (ET) domain and a C-terminal domain (CTD)^6^.

Pro-inflammatory reactions have been linked to a range of chronic situations associated with aging, including cardiovascular disease^7^, diabetes^8^, and dementia^9^. There is increasing evidence involving chronic inflammation in osteoporosis and fracture risk in adults^10^,^11^. Osteoporosis is a skeletal disorder characterized by compromised bone strength associated to an increase in fracture risk. It is most often caused by osteoclastic bone resorption that is not sufficiently compensated for by increased bone formation by osteoblasts^12^.

Organic solvents are commonly used in the pharmaceutical industry as reaction media, for product synthesis and as drug-vehicle. DMA is FDA approved as excipient and therefore widely used as drug-delivery vehicle^13^. In high risk neuroblastoma treatment DMA, at high concentration, is administered to facilitate busulfan application^14^. Busulfan is a DNA-alkylating drug applied as a myeloablative chemotherapeutic agent prior to stem cell transplantation in pediatric and adult patients suffering from neuroblastoma or other malignancies^15^. In 2009, we were the first to show that another FDA approved excipient: N-methyl pyrrolidone (NMP), is bioactive^16^,^17^ and screened other small chemicals approved by the FDA for bioactivity. From all the excipients tested, we found DMA to be epigenetically active, since it binds to bromodomains. Overall, our study shows that DMA alters osteoblast/osteoclast balance, inhibits inflammatory response and prevents estrogen depletion-induced osteoporosis. This study focuses on the potential therapeutic benefits of DMA in osteoporosis, bone regeneration and related bone diseases, and emphasizes the fact that studies where DMA or other solvents were used as controls must be analyzed with caution.

## Results

### DMA is a bromodomain ligand

**Figure 1:**
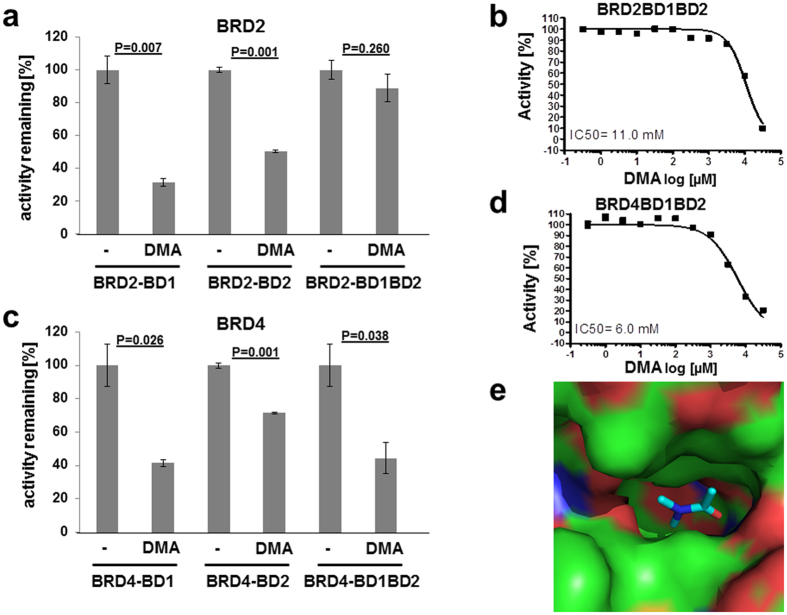
DMA acts as a low-affinity bromodomain inhibitor. DMA effect on the binding ability of bromodomains and BET bromodomains (**a**): effect 10 mM DMA on the binding ability of recombinant BRD2 using the AlphaScreening assay (n = 2 + 2). (**b**) Determination of millimolar half-maximum inhibitory concentration (IC50) for DMA on BRD2. (**c**) Effect 10 mM DMA on the binding ability of recombinant BRD4 using the AlphaScreening assay (n = 2 + 2). (**d**) Determination of millimolar half-maximum inhibitory concentration (IC50) for DMA on BRD4 binding activity. (**e**) Crystal structure of DMA (sticks) bound to BRD4-BD1 (PDB code 5HCL). P values were determined by Mann-Whitney- U Test.

A substantial portion of the bioactivity of the excipient NMP in terms of bone resorption and inflammation can be linked to its affinity to bromodomains^18^,^19^. To test DMA for its affinity to bromodomains we employed an AlphaScreen assay and studied the effect of DMA on the binding of different recombinant human bromodomains and recombinant human BET bromodomains. DMA inhibits the binding activity of BRD2 and BRD4 (Fig. 1a,c) with an IC50 value of 11 mM and 6 mM, respectively (Fig. 1b,d).

To provide further evidence of the binding of DMA to bromodomains we solved the crystal structure of the complex of DMA and the N-terminal bromodomain of BRD4 at 1.5 Å resolution (PDB code 5HCL). The X-ray structure shows that DMA occupies the acetyl lysine binding site (Fig. 1e; Table 1) and is an acceptor in a hydrogen bond with the side chain of the evolutionary conserved Asn140. Despite the fact that DMA concentrations needed for bromodomain inhibition are in the mM-range, this effect is meaningful since during busulfan administration, DMA concentrations found in serum ranges between 3.09 and 8.77 mM^20^.

### DMA for osteoporosis treatment

**Figure 2:**
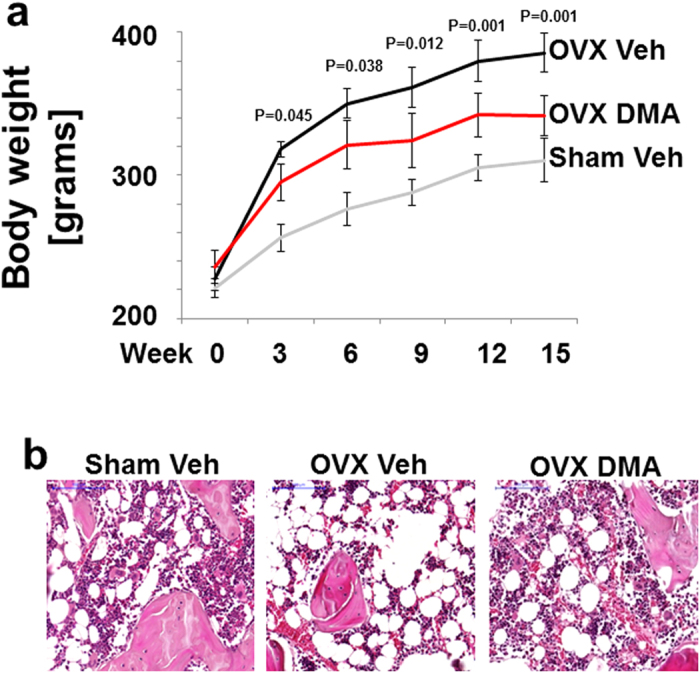
Effects of DMA rat weight and lipid accumulation within bone marrow. Body weight gain (**a**) over 15 weeks of treatment. Data are the mean ± SD values (N = 6 per group from 2 independent experiments). OVX vehicle group is significantly different from the Sham vehicle at all time points (P = 0.001). OVX vehicle group is significantly different from the OVX DMA group. P values are indicated in the figure. H&E staining (**b**) of distal femur showing the effects of ovariectomy and DMA treatment on marrow lipid accumulation. P values were determined by Mann-Whitney- U Test.

In bone, inhibition of BRD4 stops differentiation to osteoclasts^21^,^22^, the cells responsible for bone degradation. To test DMA for its potential to preserve bone tissue and for osteoporosis treatment, an ovariectomy (OVX) model in rodents was employed and DMA was administered by i.p. injections, resulting in a weekly peak concentration of 6 mM. Menopause is often accompanied by weight gain due to estrogen deficiency. We found ovariectomy promoted a significant increase in body mass (Fig. 2a) and enhanced marrow lipid accumulation within bone marrow (Fig. 2b) while DMA reversed these effects.

**Figure 3:**
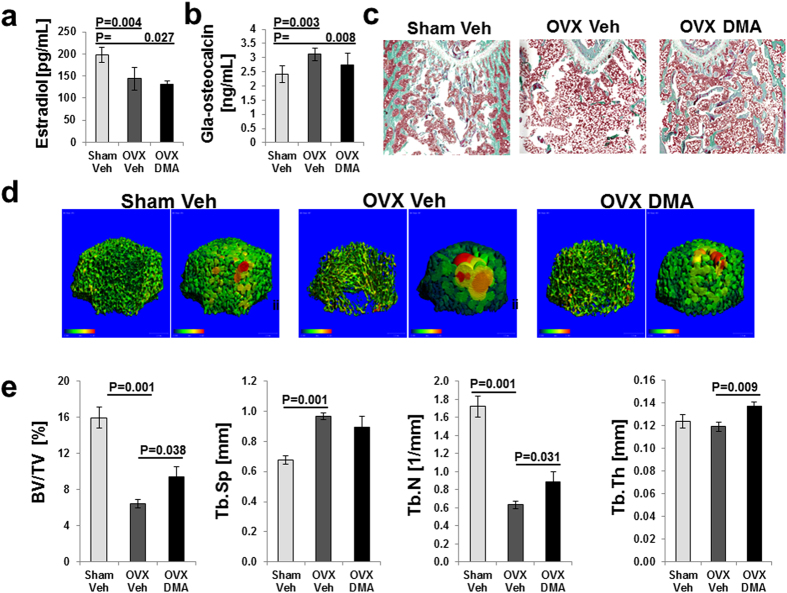
DMA prevents bone deterioration induced by estrogen depletion in rats. Changes of serum levels of estradiol after ovariectomy (**a**). The results of the Sham group were compared with OVX Veh group, and the results of the OVX DMA group were compared with the results of OVX Veh. Data are mean ± s.d. Two independent experiments were performed with 3 animals each (n = 6). Changes of serum levels of osteocalcin after ovariectomy (**b**). The results of the Sham group were compared with OVX Veh group, and the results of the OVX DMA group were compared with the results of OVX Veh. Data are mean ± s.d. Two independent experiments were performed with 3 animals each (n = 6). Goldner’s Trichrome staining of distal femur (**c**) show the effects of ovariectomy and DMA treatment on the histomorphometric parameters. Micro-CT analysis (**d**) illustrates the effects of DMA on bone loss induced by estrogen depletion. 3D image and color coded thickness map of the local diameter of the pores devoid of trabeculae. Bone parameters (**e**) were analyzed by micro-CT after 15 weeks of DMA treatment in OVX rats. Graphs represented bone volume (BV/TV), trabecular separation (Tb. Sp), trabecular number (Tb. N), and trabecular thickness (Tb. Th). Data are mean ± s.d. (n = 6 per group). P values are provided. P values were determined by Mann-Whitney- U Test.

The estradiol level in the OVX Veh group was significantly lower than in Sham Veh group (Fig. 3a). DMA was not able to rescue estradiol levels and therefore acts independent of estradiol levels. Analysis of the bone turnover marker osteocalcin revealed an increase in the OVX vehicle group compared with those in Sham vehicle group which was prevented when the OVX group was treated with DMA (Fig. 3b). Static histomorphometry analysis of femurs (Fig. 3c) revealed that DMA treatment prevents bone loss in OVX compared to the control group. The color coded thickness map of the local diameter of pores devoid of trabeculae in the femur revealed an increase in diameters of pores in the OVX sham group and confirmed that DMA treatment preserves bone architecture in the OVX-treated group (Fig. 3d).

Moreover, micro-CT scans indicate that OVX resulted in the deterioration of trabecular bone microarchitecture, supported by the reduced bone volume to total volume (BV/TV) and trabecular number (Tb.N) in comparison with the Sham group (Fig. 3e). In contrast, trabecular spacing (Tb.Sp) was significantly increased in response to OVX compared to the Sham group. DMA treatment significantly improved the microarchitecture deterioration mentioned above, but DMA was not able to reverse these parameters to a similar degree as in the Sham Veh group. Cortical bone parameters appeared unaffected.

### Bone degradation and bone regeneration

**Figure 4:**
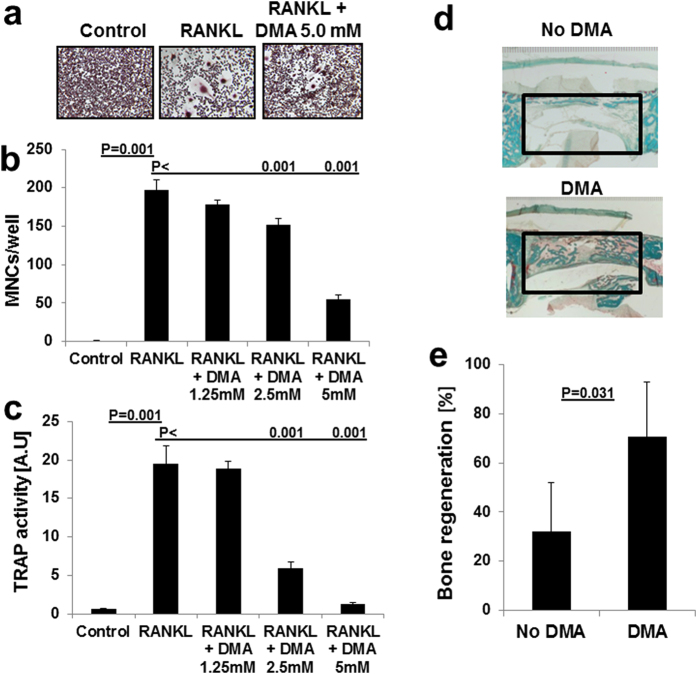
DMA inhibits osteoclast differentiation and enhances bone regeneration. Effects of DMA on osteoclast differentiation (**a**). Microscopic view of MNCs after TRAP staining. Number of multinucleated cells (MNCs) (**b**) were determined after 6 days of treatment. RAW264.7 cells were seeded on a 24-well culture plate and treated with RANKL alone or with different concentrations of DMA as indicated. After 6 days of incubation, TRAP activity was measured as described under “methods”. Data are expressed as the mean ± s.d. (n = 6) from a representative experiment. In parallel, cells were stained for TRAP after differentiation into mature osteoclasts and MNCs were counted under microscope. TRAP activity (**c**) was determined after 6 days of treatment. In parallel, cells were stained for TRAP after differentiation into mature osteoclasts and MNCs were counted under microscope. Effects of DMA on bone regeneration (**d**). Non-critical size defects were generated in calvarial bones of rabbits and treated with a biodegradable membrane with or without DMA. After 4 weeks, bone regeneration within the defect was evaluated based on the middle cross-sections. The original defects are indicated by the square. Bone appears magenta/green in the Goldner’s-Trichrome stained sections. Histomorphometric analysis (**e**) revealed significantly greater bone regeneration when the membrane contained DMA than the same membrane without DMA. (n = 6). P values were determined by Mann-Whitney- U Test.

Next we assessed the effect of DMA on bone degrading osteoclast and bone forming osteoblast differentiation and maturation. At the cellular level, DMA appears to inhibit maturation of osteoclasts induced by RANKL since in the presence of DMA less giant multinucleated osteoclasts formed (Fig. 4a,b) and cellular activity of osteoclasts determined by tartrate resistant alkaline phosphatase (TRAP) activity was decreased (Fig. 4c). To investigate the effect of DMA on bone formation we employed a guided bone regeneration model and delivered DMA via a biodegradable membrane^23^. Significantly more bone formed in this non-critical size defect when the membrane was loaded with DMA, compared to membrane alone (Fig. 4d,e). Therefore, DMA enhanced bone regeneration and bone formation *in vivo*.

**Figure 5:**
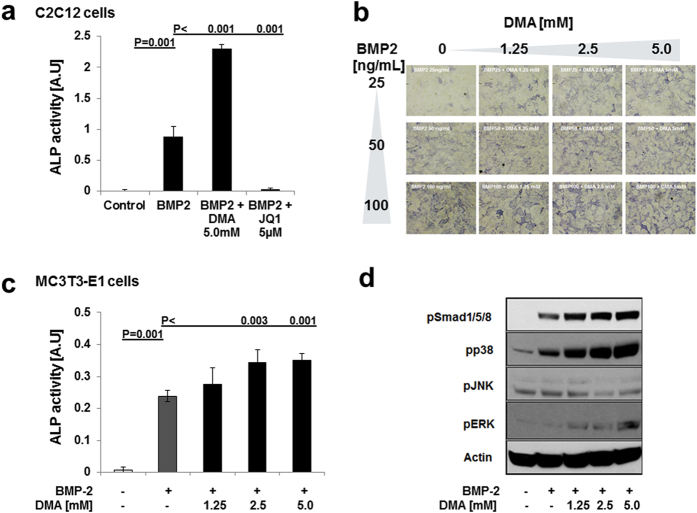
DMA improves BMP-2 induced osteoblast differentiation. Effects of DMA on osteoblast differentiation (**a**). Alkaline phosphatase activity was measured in multipotent C2C12 cells, which do not produce autologous BMP. C2C12 cells treated with BMP2 alone or with either DMA (5 mM) or JQ1 (5 μM) in the presence of BMP2. After 6 days of stimulation, ALP activity was measured (n = 6). To examine alkaline phosphatase activity histochemically, multipotent C2C12 cells stimulated with BMP-2 in the presence of DMA (**b**). Alkaline phosphatase activity was also measured in pre-osteoblast cell line, MC3T3-E1 (**c**) (N = 6). Effect of DMA on Smad and MAPK signaling (**d**). C2C12 cells were stimulated as indicated, and whole cell extracts were separated by SDS-PAGE, transferred to PVDF membranes, and probed with anti-phospho-Smad 1/5/8, anti-phospho-MAPK (Erk, Jnk and p38) antibodies. Anti-actin antibody was used as a loading control. The experiment was repeated 3 times and a representative blot is displayed (Original blots are shown in Figure S1). P values were determined by Mann-Whitney- U Test.

In an osteoblast differentiation model where multipotent cells are differentiated to osteoblasts by bone morphogenetic protein-2 (BMP2), monitored by an increase in alkaline phosphatase activity (ALP), we saw that osteoblast differentiation determined by ALP was enhanced by the low affinity bromodomain inhibitor DMA (Fig. 5a,b). The same applies to pre-osteoblastic cells (Fig. 5c). The high affinity bromodomain inhibitor JQ1, however, inhibited BMP2-induced differentiation to osteoblasts (Fig. 5a) and is known to interfere with osteoblast differentiation by inhibition of Runx2 expression^21^. The low affinity bromodomain inhibitor DMA overcomes and even enhances bone formation *in vivo* and *in vitro* (Figs 4d,e and 5a) since it not only acts as bromodomain inhibitor but also as enhancer of BMP activity by increasing Smad1/5/8 and p38 phosphorylation (Fig. 5d, S1).

### DMA and inflammation

**Figure 6:**
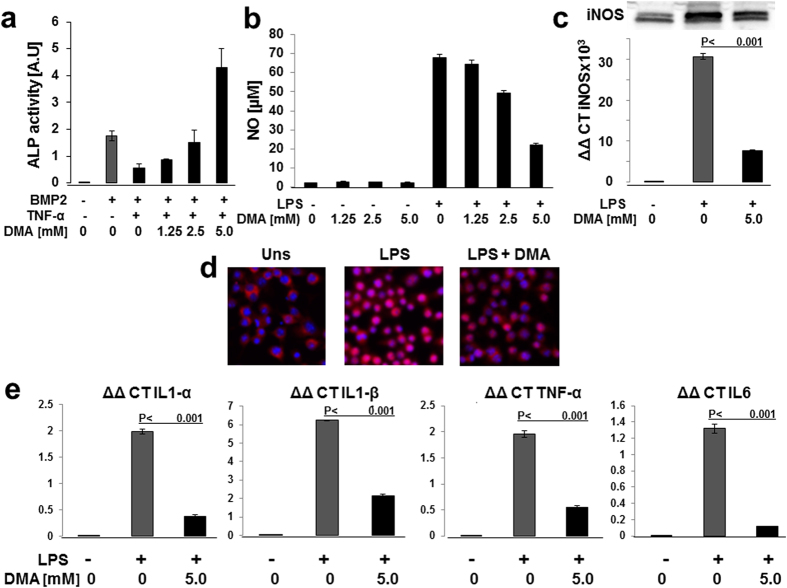
DMA opposes deleterious effect of TNF-α on osteoblast differentiation and inhibits LPS-induced inflammatory process. Effect of DMA on Alkaline phosphatase activity (**a**) was measured in multipotent C2C12 cells stimulated with BMP-2 in the presence of TNF-α and TNF-α + DMA. Effect of DMA on nitric oxide (NO) production and iNOS expression (**b**). RAW264.7 cells were incubated with 1 μg/ml LPS with or without DMA for 48 h. Data are expressed as mean ± s.d. (n = 6). For iNOS expression analysis (**c**), RAW264.7 cells were treated with LPS (1 μg/ml) in the absence or presence of DMA, total RNA (6 h after stimulation) or whole cell lysates (48 h after stimulation) were isolated. iNOS mRNA levels were determined by real-time PCR and normalized to GAPDH level and expressed as ΔΔCT. Whole cell lysates were subjected to Western blot analysis with antibody against iNOS (Original blots are shown in Figure S2). Data are expressed as mean ± s.d. (n = 6). Effect of DMA on NF-κB translocation (**d**). RAW264.7 cells were grown on 15 μ-Chamber 12 well (Ibidi, Martinsried, Germany), stimulated with LPS, and LPS after DMA pre-treatment (LPS + DMA). After 30 min stimulation, cells were fixed in 4% paraformaldehyde for 15 min at room temperature, and the subcellular localization of the NF-kB p65 subunit was shown by immunofluorescence staining. Nuclei were counterstained with Hoechst (Sigma-Aldrich). Effect of DMA on LPS-induced pro-inflammatory cytokines (**e**). RAW264.7 cells were treated with LPS (1 μg/ml) in the absence or presence of DMA, and total RNA was isolated 6 h after treatment. IL-1α, IL-β, TNF-α, and IL-6 mRNA levels were determined by real-time PCR and normalized to GAPDH level Results are expressed as ΔΔCT. Data are expressed as mean ± s.d. (n = 6) and P values provided. P values were determined by Mann-Whitney- U Test.

To address inflammation, as the third aspect of osteoporosis, we investigated the effect of DMA on the TNF-α inhibition of BMP-induced osteogenesis. BMP2-induced ALP activity was decreased by TNF-α treatment. Pretreatment with DMA not only abolished the inhibitory effect of TNF-α but also potentiated the effect of BMP-2 dose dependently (Fig. 6a).

To further analyze the inflammatory process, we stimulated RAW264.7 macrophages with lipopolysaccharide (LPS), causing an accumulation of nitric oxide (NO), a characteristic feature of activated macrophages (Fig. 6b). DMA alone had no effect on NO production. DMA pretreatment, however, suppressed LPS-induced NO production in a dose-dependent manner. The change of iNOS expression, the enzyme responsible for NO production, was examined and iNOS was induced with LPS treatment and this induction was diminished substantially by DMA both at the protein and mRNA level (Fig. 6c).

Since nuclear factor-κB (NF-κB) is involved in the transcriptional regulation of cytokines and targeted by bromodomain inhibitors, we examined the nuclear translocation of p65 which tightly linked to NFκB activation. In essence, we analyzed the cellular location changes of NF-κB/p65 after LPS stimulation with or without DMA pre-treatment (Fig. 5d). In unstimulated cells NF-κB/p65 was located in the cytoplasm. In LPS-treated cells NF-κB/p65 translocated into the nucleus and pre-treatment with DMA partially suppressed the LPS-induced p65 translocation, suggesting that DMA inhibits the LPS-induced NF-κB activation. Macrophages orchestrate the immune response, hematopoiesis, and other homeostatic processes via cytokine expression and release. mRNA expression of IL-1α, IL-1β, IL-6 and TNF-α were markedly up-regulated after LPS stimulation, however, significantly reduced by DMA pretreatment (Fig. 6e).

## Discussion

Therapeutic agents targeting protein expression on the epigenetic level have shown encouraging antitumor activity. Bromodomain inhibitors decrease the expression of oncogenes like myc and Runx2^21^,^24^,^25^,^26^,^27^ and are presently being tested in clinical trials. Recent studies demonstrated that JQ1, a potent inhibitor of BRD2, BRD3, BRD4, and BET proteins, beside its antitumor activity suppress inflammation by a reduction of the inflammatory cytokine release, bone destruction by the inhibition of osteoclast maturation and bone formation by the inhibition of osteoblast maturation^21^,^22^. The activities of JQ1 in suppression of inflammation and the inhibition of osteoclast maturation were sufficient for a short term treatment of periimplantitis^22^. For long-term treatment of osteoporosis, however, the use of JQ1 could compromise the treatment due to its inhibiting effect on osteoblast maturation^21^.

In the present study, we showed that DMA preserves bone mass and architecture in a postmenopausal osteoporosis model based on estrogen deficiency (Fig. 3) and thus has the potential to treat and prevent osteoporosis. DMA was proved to be a ligand for bromodomains (Fig. 1) and shares with JQ1 the potential to reduce the inflammatory cytokine release and to inhibit osteoclast maturation (Fig. 4a–c). In contrast to the high affinity bromodomain ligand JQ1, the low affinity ligand DMA, however, was shown here to enhance osteoblast maturation induced by BMP-2 *in vitro* and bone regeneration *in vivo* even without the application of recombinant BMP-2 (Fig. 4d,e and 5). Although BRD4 bromodomain inhibition is associated with the reduction of Runx2 expression needed for osteoblast differentiation, the lower affinity of DMA to BRD4 and the additional activity of enhancing the kinase activity of the BMP-2 receptor for Smads and p38 overcomes the reduced Runx2 expression. At the bottom line, DMA even enhances bone regeneration and osteoblast maturation *in vivo* (Fig. 4d,e).

Chronic inflammation plays an important role in the development of several diseases such as rheumatoid arthritis (RA) and osteoporosis. Both pathologies are associated with increased risk of fractures and systemic bone loss. Since bone loss is related to the activation of TNF-α system, it can be hypothesized that TNF-α directly controls osteoblast survival and/or function in addition to its induction of osteoclast differentiation leading to excess of bone resorption^28^. This can also explain the association between inflammation and osteoporosis^29^. The suppressive effect of DMA on inflammation has been discovered and patented in 1962^30^. In our study we propose the molecular mechanism of this effect and suggest that DMA exerts its anti-inflammatory activity as competitor of bromodomain binding (Fig. 1). The effect of DMA on the inflammatory process (Fig. 6) in particular mediated by NFκB-dependent mechanisms is not unexpected since inhibitors of bromodomain have shown therapeutic promise in several diseases including inflammation-related ones^31^. For the long-term treatment of chronic inflammatory diseases DMA has the advantage of non-interference with bone formation and regeneration, for acute inflammatory diseases like sepsis, high affinity bromodomain inhibitors like JQ1 are certainly more effective. Moreover, DMA reduced the weight of the ovariectomized animals and the fat content in the marrow which points to the potential of DMA to be used for adiposity treatment (Fig. 2). The same was reported for JQ1 with weight loss being the sole side effect of JQ1 application for contraception in male mice^32^.

The preclinical data presented in this study establish the potential of low affinity bromodomain inhibitors like DMA for long term treatments as needed in osteoporosis, adiposity, or chronic inflammation related diseases. DMA is FDA approved and presents no toxic effect in human. In fact, it has been reported that DMA, when used as a vehicle for intravenous busulfan, does not produce adverse effects in pediatric oncologic patients at doses even higher than those shown to suppress inflammation^33^. The concentration, however, needed for effect is in the millimolar range. Therefore, clinical long term studies will be required to understand these risks in more detail.

We conclude that DMA functions as bromodomain inhibitor and BMP enhancer and displays a potential role as an anti-osteoporotic drug based on its triple effect: inhibition of osteoclastogenesis, enhancement of osteoblastogenesis, in combination with its anti-inflammatory activity. Agents that modulate or inhibit epigenetic modifications have shown great potential in inhibiting the progression of many diseases. Given the effectiveness of DMA, there is great potential for the development of novel therapeutics with DMA as direct or adjuvant therapeutic compound for bone related diseases.

## Methods

### AlphaScreening assay

AlphaScreening assay was performed as previously described^19^. The percent inhibition in the presence of DMA was calculated according to the following equation: % inhibition =100 - % activity. The IC50 value was determined by the concentration causing a half-maximal percent activity.

### Bromodomain expression and purification

Briefly, His-tagged BRD4 (residues 42–168, BRD4-BD1) was expressed in Escherichia coli BL21 (DE3) cells upon induction with isopropyl thio-beta-D-galactoside (IPTG, final concentration 0.1 mM) for 16 h at 18 °C. Bacteria were lysated and the His-tagged proteins were purified on HisTrap columns (GE Healthcare) and eluted using a step gradient of imidazole. The poly-Histidine tags were removed by overnight incubation with His-tagged tobacco etch virus (TEV) protease purified in-house. A size-exclusion chromatography step (HiLoad 16/600 Superdex75 column) and a Ni-affinity chromatography step were subsequently performed to finally purify the cleaved bromodomains. Samples were then concentrated, flash frozen and stored at −80 °C.

### X-ray crystallography, Data Collection, and Structure Determination (PDP code 5HCL)

Apo crystals of the BRD4-BD1 bromodomain were grown at 4 °C using the hanging drop vapor diffusion method under the condition 0.2 M Sodium Nitrate, 20% PEG 3350, 10% ethylene glycol. The apo crystals were then soaked with 10% (V/V) DMA overnight and flash-frozen for measurements. Data sets were collected on a PILATUS 16 M detector at the Swiss Light Source beamline X06SA of the Paul Scherrer Institute (Villigen, Switzerland) and indexed, integrated and scaled with the XDS^34^ and CCP4 programs^35^. The structures were solved by molecular replacement with PHASER^36^ using the BRD4 structure (PDB entry 4PCI) as a search model and refined with PHENIX^37^. The atomic coordinates and structure factors of BRD4 in complex with DMA have been deposited into the Protein Data Bank as entry 5HCL.

Data collection and refinement statistics are given (Table 1).

### Animal experiments

All animal procedures were approved by the Animal Ethics Committee of the local authorities (Canton Zurich, 40/2012; 108/2012) and performed in accordance with the ethics criteria contained in the bylaws of the Institutional Animal Care and Use Committee. After the acclimatization period, the animals (female, 15 weeks old, Sprague Dawley) were randomly bilaterally ovariectomized (OVX, *n* = 12) or sham-operated (Sham, *n* = 6). One week after surgery, the OVX rats were divided into 2 groups: OVX with vehicle (OVX Veh, n = 6) and OVX with DMA (OVX DMA, n = 6). Treatment was initiated 1 week after OVX and lasted for 15 weeks. DMA was administered once a week via i.p. injections (92 μl DMA plus 108 μl PBS per 100 gr. rat). The n of 6 derived from 2 independent experiments with n = 3 each. Sample size was determined by power analysis. Whole blood sample was collected via abdominal aorta puncture immediately following sacrifice by CO_2_ asphyxiation. Then, a serum specimen was harvested after centrifugation (2000 rpm for 20 min) and stored at −80 °C until further analysis. Femurs were dissected and the adherent tissue removed before placing the samples in 70% ethanol and later used for micro-CT analysis and measurement of bone parameters. Bone regeneration was tested in a guided bone regeneration model at the calvarial bone of 6 rabbits (female, 26 week old, New Zealand white rabbit) as described earlier^23^. DMA loading of the membrane was performed by vapor deposition to yield 10% weight increase^23^. Sample size was determined by power analysis.

### Histology

Paraffin- and methacrylate-based histology was performed as previously described^19^.

### Serological Analysis of Bone Markers

Serum markers were analyzed to monitor the treatment effect on bone physiology as previously described^19^.

### Microcomputer Tomography Analysis (μCT)

The rat femur samples were measured with a cone-beam microCT (μCT 100, SCANCO MEDICAL AG, Brüttisellen, Switzerland) as previously described^19^. The parameters were: bone volume over total volume (BV/TV), trabecular number (Tb.N), trabecular separation (Tb.Sp), and trabecular thickness (Tb.Th).

### Cell Cultures

RAW264.7, C2C12 and MC3T3-E1 cells were purchased from American Type Culture Collection (ATCC). Cells were cultured in Dulbecco’s modified Eagle’s medium (DMEM) (C2C12 and RAW264.7) or alpha-minimal essential medium (MC3T3-E1) and treated as described previously^38^,^39^. All cell experiments were performed 2 times independently with an n = 3 to yield an overall n of 6. Sample size for cells is fixed at n = 6 based on our experience. All cell lines were mycoplasma tested via pcr (Promokine Kit).

### ALP activity assay and ALP staining

Alkaline phosphatase (ALP) was used as a marker of osteoblastic differentiation. ALP activity and ALP staining were evaluated as described previously^17^. Briefly, cells were seeded in 24-well plates (n = 6 per group). One day later, cells were incubated with BMP2 in the presence or absence of DMA for 6 days. After incubation, ALP activity was measured on whole cell extract using p-nitrophenylphosphate (Sigma) as a substrate. To examine alkaline phosphatase activity histochemically, cells were fixed for 10 min with 3.7% formaldehyde at room temperature. After washing with PBS, the cells were stained as described in^40^. Images of stained cells were captured with a CDD camera.

### Western blot analysis

Treated cells were rapidly frozen in liquid nitrogen and stored at − 80 °C until used for analysis as previously described^38^,^39^.

### TRAP activity, TRAP staining, Nitrite determination, Quantitative real-time PCR (qPCR), and Nuclear translocation of p65

All these methodologies were performed as previously described^16^,^41^.

### Statistical analysis

All statistical analysis were performed with IBM SPSS statistics 22. Results are expressed as the mean ± SD and were compared by the non-parametric Kruskal-Wallis-Test followed by the Mann-Whitney- U Test. Results were considered significantly different for P < 0.05.

## Additional Information

**How to cite this article**: Ghayor, C. *et al*. N,N Dimethylacetamide a drug excipient that acts as bromodomain ligand for osteoporosis treatment. *Sci. Rep.* **7**, 42108; doi: 10.1038/srep42108 (2017).

**Publisher's note:** Springer Nature remains neutral with regard to jurisdictional claims in published maps and institutional affiliations.

## Supplementary Material

Supplemental Information

## Figures and Tables

**Table 1:** Atomic coordinates and structure factors of BRD4 in complex with DMA as deposited into the Protein Data Bank as entry 5HCL.

Compound	DMA
Bromodomain	BRD4
**Data collection**	
Space group	P 21 21 21
Unit cell	
a (Å)	37.62
b (Å)	43.83
c (Å)	78.46
alpha (°)	90
beta (°)	90
gamma (°)	90
Resol. range (Å)	39.23–1.50
Unique reflections	21250 (3002)
<I/σ(I)>	42.5 (28.2)
R merge	0.031 (0.045)
Completeness (%)	99.2 (98.1)
Multiplicity	6.5 (6.2)
**Refinement**	
Resol. range (Å)	39.23–1.50
R factor/R free	14.52/17.54
Mean B factors (A2)	12.50
RMS bonds (Å)	0.0057
RMS angles (°)	0.820

## References

[b1] Zeng L. . Selective Small Molecules Blocking HIV-1 Tat and Coactivator PCAF Association. Journal of the American Chemical Society 127, 2376–2377, doi: 10.1021/ja044885g (2005). 15724976

[b2] Filippakopoulos P. & Knapp S. Targeting bromodomains: epigenetic readers of lysine acetylation. Nat Rev Drug Discov 13, 337–356, doi: 10.1038/nrd4286, http://www.nature.com/nrd/journal/v13/n5/abs/nrd4286.html#supplementary-information (2014). 24751816

[b3] Nicodeme E. . Suppression of inflammation by a synthetic histone mimic. Nature 468, 1119–1123, doi: http://www.nature.com/nature/journal/v468/n7327/abs/nature09589.html#supplementary-information (2010). 21068722 10.1038/nature09589 PMC5415086

[b4] Filippakopoulos P. . Selective inhibition of BET bromodomains. Nature 468, 1067–1073, doi: 10.1038/nature09504 (2010). 20871596 PMC3010259

[b5] Huang B., Yang X. D., Zhou M. M., Ozato K. & Chen L. F. Brd4 coactivates transcriptional activation of NF-kappaB via specific binding to acetylated RelA. Mol Cell Biol 29, 1375–1387, doi: 10.1128/MCB.01365-08 (2009). 19103749 PMC2643823

[b6] Denis G. V., Nikolajczyk B. S. & Schnitzler G. R. An emerging role for bromodomain-containing proteins in chromatin regulation and transcriptional control of adipogenesis. FEBS letters 584, 3260–3268, doi: 10.1016/j.febslet.2010.05.030 (2010). 20493850 PMC2914217

[b7] Pai J. K. . Inflammatory markers and the risk of coronary heart disease in men and women. N Engl J Med 351, 2599–2610, doi: 10.1056/NEJMoa040967 (2004). 15602020

[b8] Hu F. B., Meigs J. B., Li T. Y., Rifai N. & Manson J. E. Inflammatory markers and risk of developing type 2 diabetes in women. Diabetes 53, 693–700 (2004). 14988254 10.2337/diabetes.53.3.693

[b9] Weaver J. D. . Interleukin-6 and risk of cognitive decline: MacArthur studies of successful aging. Neurology 59, 371–378 (2002). 12177370 10.1212/wnl.59.3.371

[b10] Barbour K. E. . Inflammatory markers and risk of hip fracture in older white women: the study of osteoporotic fractures. J Bone Miner Res 29, 2057–2064, doi: 10.1002/jbmr.2245 (2014). 24723386 PMC4336950

[b11] Cauley J. A. . Inflammatory markers and incident fracture risk in older men and women: the Health Aging and Body Composition Study. J Bone Miner Res 22, 1088–1095, doi: 10.1359/jbmr.070409 (2007). 17419681

[b12] Rodan G. A. & Martin T. J. Therapeutic approaches to bone diseases. Science 289, 1508–1514 (2000). 10968781 10.1126/science.289.5484.1508

[b13] Oechtering D., Boos J. & Hempel G. Monitoring of N,N-dimethylacetamide in children during i.v.-busulfan therapy by liquid chromatography–mass spectrometry. Journal of Chromatography B 838, 129–134, doi: 10.1016/j.jchromb.2006.04.034 (2006). 16725388

[b14] Maris J. M., Hogarty M. D., Bagatell R. & Cohn S. L. Neuroblastoma. Lancet 369, 2106–2120, doi: 10.1016/S0140-6736(07)60983-0 (2007). 17586306

[b15] London W. B. . Clinical and biologic features predictive of survival after relapse of neuroblastoma: a report from the International Neuroblastoma Risk Group project. Journal of clinical oncology: official journal of the American Society of Clinical Oncology 29, 3286–3292, doi: 10.1200/JCO.2010.34.3392 (2011). 21768459 PMC3158599

[b16] Ghayor C. . Inhibition of osteoclast differentiation and bone resorption by N-methylpyrrolidone. J Biol Chem 286, 24458–24466, doi: 10.1074/jbc.M111.223297 (2011). 21613210 PMC3129225

[b17] Miguel B. S. . N-methyl pyrrolidone as a potent bone morphogenetic protein enhancer for bone tissue regeneration. Tissue Eng Part A 15, 2955–2963, doi: 10.1089/ten.TEA.2009.0009 (2009). 19320543

[b18] Gjoksi B., Ghayor C., Bhattacharya I., Zenobi-Wong M. & Weber F. E. The bromodomain inhibitor N-methyl pyrrolidone reduced fat accumulation in an ovariectomized rat model. Clin Epigenetics 8, 42, doi: 10.1186/s13148-016-0209-2 (2016). 27110299 PMC4840488

[b19] Gjoksi B. . The epigenetically active small chemical N-methyl pyrrolidone (NMP) prevents estrogen depletion induced osteoporosis. Bone 78, 114–121, doi: 10.1016/j.bone.2015.05.004 (2015). 25959414

[b20] Trame M. N., Bartelink I. H., Boos J., Boelens J. J. & Hempel G. Population pharmacokinetics of dimethylacetamide in children during standard and once-daily IV busulfan administration. Cancer Chemother Pharmacol 72, 1149–1155, doi: 10.1007/s00280-013-2284-9 (2013). 24036908

[b21] Lamoureux F. . Selective inhibition of BET bromodomain epigenetic signalling interferes with the bone-associated tumour vicious cycle. Nat Commun 5, 3511, doi: 10.1038/ncomms4511 (2014). 24646477

[b22] Meng S. . BET Inhibitor JQ1 Blocks Inflammation and Bone Destruction. J Dent Res 93, 657–662, doi: 10.1177/0022034514534261 (2014). 24799421 PMC4107547

[b23] Karfeld-Sulzer L. S. . Comparative study of NMP-preloaded and dip-loaded membranes for guided bone regeneration of rabbit cranial defects. Journal of tissue engineering and regenerative medicine, doi: 10.1002/term.1926 (2014). 24919954

[b24] Dawson M. A. & Kouzarides T. Cancer epigenetics: from mechanism to therapy. Cell 150, 12–27, doi: 10.1016/j.cell.2012.06.013 (2012). 22770212

[b25] Delmore J. E. . BET bromodomain inhibition as a therapeutic strategy to target c-Myc. Cell 146, 904–917, doi: 10.1016/j.cell.2011.08.017 (2011). 21889194 PMC3187920

[b26] Roe J. S., Mercan F., Rivera K., Pappin D. J. & Vakoc C. R. BET Bromodomain Inhibition Suppresses the Function of Hematopoietic Transcription Factors in Acute Myeloid Leukemia. Mol Cell 58, 1028–1039, doi: 10.1016/j.molcel.2015.04.011 (2015). 25982114 PMC4475489

[b27] Blyth K. . Runx2 and MYC Collaborate in Lymphoma Development by Suppressing Apoptotic and Growth Arrest Pathways *In vivo*. Cancer Research 66, 2195–2201, doi: 10.1158/0008-5472.can-05-3558 (2006). 16489021

[b28] Kudo O. . Proinflammatory cytokine (TNFalpha/IL-1alpha) induction of human osteoclast formation. J Pathol 198, 220–227, doi: 10.1002/path.1190 (2002). 12237882

[b29] Huang H. . Dose-specific effects of tumor necrosis factor alpha on osteogenic differentiation of mesenchymal stem cells. Cell Prolif 44, 420–427, doi: 10.1111/j.1365-2184.2011.00769.x (2011). 21951285 PMC6495272

[b30] Glenn E. M. Topical pharmaceutical formulations containing n, n-dimethylacetamide as an anti-inflammatory ingredient Patent: US 3068145A (1962).

[b31] Mirguet O. . Naphthyridines as novel BET family bromodomain inhibitors. ChemMedChem 9, 580–589, doi: 10.1002/cmdc.201300259 (2014). 24000170

[b32] Matzuk M. M. . Small-molecule inhibition of BRDT for male contraception. Cell 150, 673–684, doi: 10.1016/j.cell.2012.06.045 (2012). 22901802 PMC3420011

[b33] Hempel G. . Cytotoxicity of dimethylacetamide and pharmacokinetics in children receiving intravenous busulfan. J Clin Oncol 25, 1772–1778, doi: 10.1200/JCO.2006.08.8807 (2007). 17470868

[b34] Kabsch W. Automatic processing of rotation diffraction data from crystals of initially unknown symmetry and cell constants. Journal of Applied Crystallography 26, 795–800, doi: 10.1107/S0021889893005588 (1993).

[b35] Winn M. D. . Overview of the CCP4 suite and current developments. Acta Crystallographica Section D 67, 235–242, doi: 10.1107/S0907444910045749 (2011). PMC3069738 21460441

[b36] McCoy A. J. . Phaser crystallographic software. Journal of Applied Crystallography 40, 658–674, doi: 10.1107/S0021889807021206 (2007). 19461840 PMC2483472

[b37] Adams P. D. . PHENIX: building new software for automated crystallographic structure determination. Acta Crystallographica Section D 58, 1948–1954, doi: 10.1107/S0907444902016657 (2002). 12393927

[b38] Ghayor C., Ehrbar M., San Miguel B., Gratz K. W.& Weber F. E. cAMP enhances BMP2-signaling through PKA and MKP1-dependent mechanisms. Biochem Biophys Res Commun 381, 247–252, doi: 10.1016/j.bbrc.2009.02.032 (2009). 19217886

[b39] Ghayor C., Rey A. & Caverzasio J. Prostaglandin-dependent activation of ERK mediates cell proliferation induced by transforming growth factor beta in mouse osteoblastic cells. Bone 36, 93–100, doi: 10.1016/j.bone.2004.10.007 (2005). 15664007

[b40] Katagiri T. . Bone morphogenetic protein-2 converts the differentiation pathway of C2C12 myoblasts into the osteoblast lineage. J Cell Biol 127, 1755–1766 (1994). 7798324 10.1083/jcb.127.6.1755 PMC2120318

[b41] Ghayor C., Gjoksi B., Siegenthaler B. & Weber F. E. N-methyl pyrrolidone (NMP) inhibits lipopolysaccharide-induced inflammation by suppressing NF-kappaB signaling. Inflamm Res 64, 527–536, doi: 10.1007/s00011-015-0833-x (2015). 26047594

