# Supplementary material for: N,N Dimethylacetamide a drug excipient that acts as bromodomain ligand for osteoporosis treatment
Source: Sci Rep. 2017 Feb 8;7:42108. doi: 10.1038/srep42108 (PMC5296751; doi:10.1038/srep42108)
Supplement: Supplemental Information [file srep42108-s1.pdf]

Supplementary information

N,N Dimethylacetamide a drug excipient that acts as bromodomain ligand for osteoporosis treatment.

Chafik Ghayor[1], Bebeka Gjoksi[1,2], Jing Dong[3], Barbara Siegenthaler[1,4], Amedeo Caflisch[3], Franz E. Weber[1,2,4,] *

[1]Oral Biotechnology & Bioengineering, Center for Dental Medicine/MKG, University of Zürich, Switzerland.

[2]Zurich Center for Integrative Human Physiology (ZIHP), University of Zurich, Switzerland

[3]Dept. of Biochemistry, University of Zurich, Switzerland,

[4]CABMM, Center for Applied Biotechnology and Molecular Medicine, University of Zurich, Zurich, Switzerland.

*****Correspondence: franz.weber@zzm.uzh.ch

Supplemental Figure S1

Full-length blots of Figure 5d

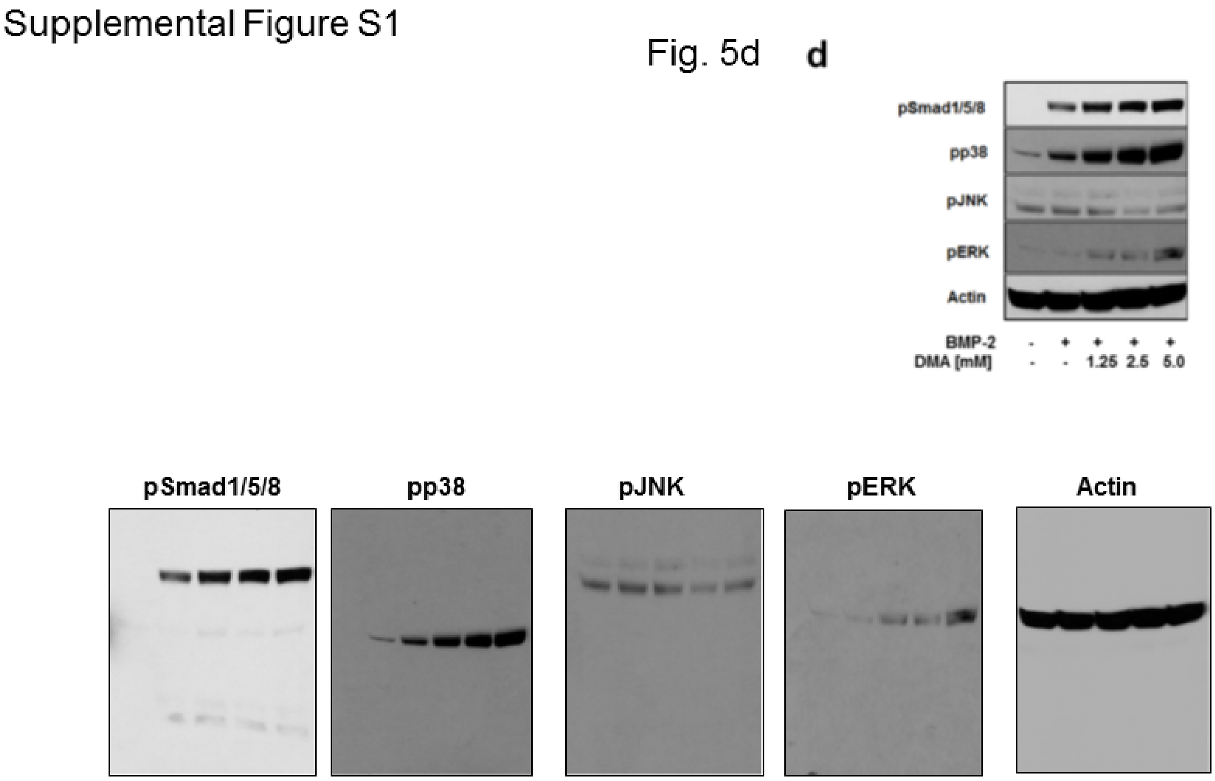

Proteins were separated on a 4–20 % precast polyacrylamide gel (Bio-Rad), and transferred to PVDF membrane using Trans-Blot Turbo Transfer System (Bio-Rad). The proteins were detected by using the appropriate primary antibodies followed by horseradish peroxidase (HRP)-coupled secondary antibody. The membranes were washed, treated with the ECL reagent, and exposed to X-ray films. Filters that were reprobed were stripped according to the manufacturer's protocol.

Supplemental Figure S2

Full-length blots of Figure 6c

Supplemental Figure S2

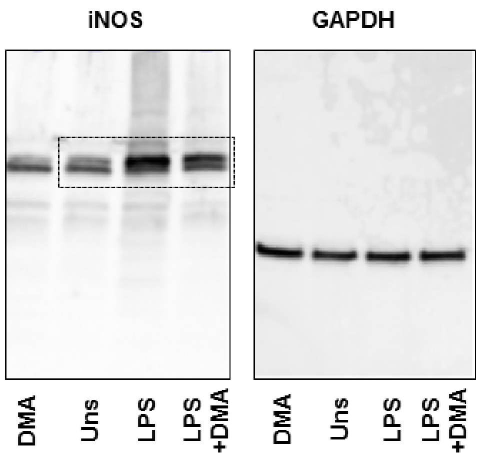

Fig. 6c

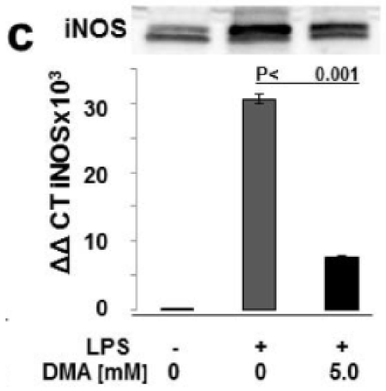

Proteins were separated on a 4–20 % TGX stain-free precast gel (Bio-Rad), and transferred to PVDF membrane using Trans-Blot Turbo Transfer System (Bio-Rad). The proteins were detected by using the appropriate primary antibodies followed by horseradish peroxidase (HRP)-coupled secondary antibody. The membranes were washed and incubation in Clarity western ECL substrate chemiluminescent detection reagent (Bio-Rad) for 5 min prior to image acquisition. The chemiluminescent blots were imaged with the ChemiDoc MP imager (Bio-Rad).
For the figure 6c, we only used unstimulated, LPS and LPS+DMA groups (framed in dotted lines).
